# Association Between Social Participation, Physical Activity, and Intrinsic Capacity Decline: Empirical Evidence from the CHARLS

**DOI:** 10.3390/healthcare14070936

**Published:** 2026-04-03

**Authors:** Lin Hu, Jing Tan, Chuan Pu

**Affiliations:** School of Public Health, Chongqing Medical University, Chongqing 400016, China; 2023121659@stu.cqmu.edu.cn (L.H.); 2023121696@stu.cqmu.edu.cn (J.T.)

**Keywords:** social participation, physical activity, older adults, intrinsic capacity, healthy aging

## Abstract

**Highlights:**

**What are the main findings?**

- Social participation levels exhibit a dose–response relationship with the risk of declining intrinsic capacity.
- Physical activity levels exhibit a U-shaped relationship with the risk of declining intrinsic capacity.

**What are the implications of the main findings?**

- Social participation plays a positive role in protecting intrinsic capacity.
- A nonlinear threshold relationship suggests that moderate physical activity yields the greatest benefits for intrinsic capacity protection.

**Abstract:**

**Objectives**: The reduction in intrinsic capacity significantly impacts the functional abilities of older individuals, and is strongly linked to adverse health consequences. Safeguarding and enhancing an elderly person’s intrinsic capacity can lead to better life quality and improved social well-being. This research seeks to explore the relationships between social engagement, physical activity, and the likelihood of decline in intrinsic capacity among the elderly in China. **Methods**: Utilizing the CHARLS data from 2015, individuals with incomplete information were removed from our study. Our analysis included a total of 3502 samples. Social participation and physical activity were assessed through self-reported surveys. The evaluation of intrinsic capacity, based on WHO criteria, thoroughly examined participants in five areas: mobility, sensory functions, vitality, mental health and cognitive abilities. The links between social participation, physical activity and intrinsic capacity decline were revealed through logistic regression. Restricted cubic splines (RCS) were employed as a statistical model, exploring the relationships between dose and response. Interaction analysis was used to examine the interaction between social participation and physical activity. Analyses of subgroups facilitated the evaluation of variations based on factors including age, gender, duration of sleep, and chronic disease numbers. **Results**: In contrast to the low-level group, individuals with moderate to high degrees of social participation (OR = 0.80, *p* = 0.012; OR = 0.56, *p* < 0.001) and those with moderate to high levels physical activity (OR = 0.72, *p* = 0.019; OR = 0.74, *p* = 0.016) demonstrated a notably lower risk of decline in intrinsic capacities. A negative correlation was identified in a dose-response manner between social participation and the risk of IC decline. A U-shaped relationship was established between physical activity levels and the risk of intrinsic capacity decline. The fully adjusted interaction model showed that no significant interaction was observed between social participation and physical activity (*p* = 0.778). Furthermore, subgroup analyses showed that these associations remained generally consistent across older adults of different age groups, genders, sleep duration, and numbers of chronic diseases. **Conclusions**: In order to slow down the deterioration of intrinsic capacity in older adults in China, it may be beneficial to focus on sustaining elevated levels of social participation and engaging in moderate physical activity. Higher levels of social participation are associated with a lower risk of experiencing a decline in intrinsic capacity, whereas both insufficient and excessive physical activity are associated with an increased risk of intrinsic capacity decline.

## 1. Introduction

Life expectancy is on the rise, and the fertility rate is declining, which is having a great impact on global demographic patterns [1,2]. The proportion of those aged 60 and above is increasing and is expected to reach 16% by the year 2050 [3]. Some common age-related concerns, such as chronic diseases and frailty, deteriorate functional ability, which may ultimately result in diminished independence, social isolation and a reduced life quality for older individuals [4,5]. Healthy aging is thus now considered a major global priority for protecting the growing aged population. The World Health Organization (WHO) has defined intrinsic capacity as a crucial indicator for assessing healthy aging [6]. The ultimate goal is to promote the health of seniors and support high-quality longevity.

Intrinsic capacity (IC) stands for the entire physical and mental capacity that an individual can utilize at any given moment. It consists of five dimensions: locomotion, sensory functions (including vision and hearing), vitality, cognitive abilities, and psychological function [7]. It is often employed to evaluate an individual’s ability to manage challenges, maintain health, and live autonomously in their later years. Research indicates that IC generally declines with advancing age [8]. IC decline has also been linked to various negative health outcomes in older individuals, including frailty [9], falls [10], disability [11], institutionalization [12], and mortality [13]. Furthermore, IC is affected by factors including demographic background, health-related behaviors, and living environments [14,15,16]. A decline in IC severely restricts the functional performance and life quality for older adults, not only increasing caregiving burden on individuals and families but also potentially driving up societal healthcare expenditures. This presents a great danger to social–economic development and public health systems. Recent meta-analyses revealed that approximately 66.0% of Chinese older people experience IC decline [17]. Moreover, the worldwide prevalence of older adults with IC impairment together with other diseases is as high as 55.0% [18]. Thus, early assessment and timely intervention become particularly important in slowing down IC decline, better supporting older adults’ ability to live independently, enhancing quality of life in later years, and promoting healthy aging.

Activity participation is recognized as an essential element of healthy aging and one potential factor influencing IC. It typically encompasses social participation (SP), such as socializing with friends or groups, and physical activity (PA), such as exercise or a structured physical training program [19]. Social participation and physical activity are two conceptually distinct yet potentially complementary behavioral dimensions [20,21]. Social participation primarily reflects individuals’ engagement in interpersonal, community, and societal activities, whereas physical activity mainly refers to behaviors related to bodily movement and energy expenditure. Although these two dimensions emphasize different aspects, both play positive roles in healthy aging, and interventions targeting both dimensions simultaneously have shown potential to improve health-related outcomes [22,23]. Existing evidence further suggests that social participation and physical activity may be linked to health through partly different pathways: social participation is more closely related to social connectedness, psychosocial support, and cognitive stimulation, whereas physical activity is more strongly associated with locomotor function, vitality, and the maintenance of overall physical functioning [24,25,26,27,28,29,30]. Therefore, it is necessary to examine the independent and joint associations of social participation and physical activity with intrinsic capacity impairment within the same analytical framework.

Recent studies have emphasized the connections between both social participation and physical activity and IC, showcasing the significant impact of these factors on its preservation. Research indicates that older individuals who are socially isolated are more likely to experience IC decline [31]. Further, social participation mediates the association between internet use and IC [32,33]. The research further suggested that physical activity is linked to some domains of IC (locomotion, vitality, and cognition), albeit in an inconsistent fashion [34,35], which may be due to differing study design, populations, or methodologies. On the other hand, recent research revealed that older adults with lower IC might face increased challenges in sustaining independent participation in daily social and physical activities due to a higher risk of limitations in ADL [36,37]. It is noteworthy that current research has focused on the correlations or mediating effects between SP and PA with IC. Far less attention has been paid to how these factors might be linked through dose–response relationships. In fact, nonlinear relationships may exist between these variables, and the associations may differ by age and gender. Accordingly, social participation and physical activity need to be investigated in relation to IC decline risk in the elderly to enrich relevant studies and provide scientific evidence for future studies in the field.

This research aims to delve into the associations between both social participation and physical activity and the risk of IC decline among older people, utilizing the third wave of data from the CHARLS. Meanwhile, it attempts to evaluate potential nonlinear relationships, which could offer good evidence for future research and intervention management to delay the decline of IC and promote healthy aging.

## 2. Materials and Methods

### 2.1. Study Design and Participants

The China Health and Retirement Longitudinal Study (CHARLS) is conducted by the National School of Development at Peking University. It aims to collect high-quality, multidimensional microdata concerning individuals aged 45 and above in China [38]. This study received ethical approval (IRB00001052-11015). All participants signed their informed consent. Data is available for download from the CHARLS platform.

**Figure 1:**
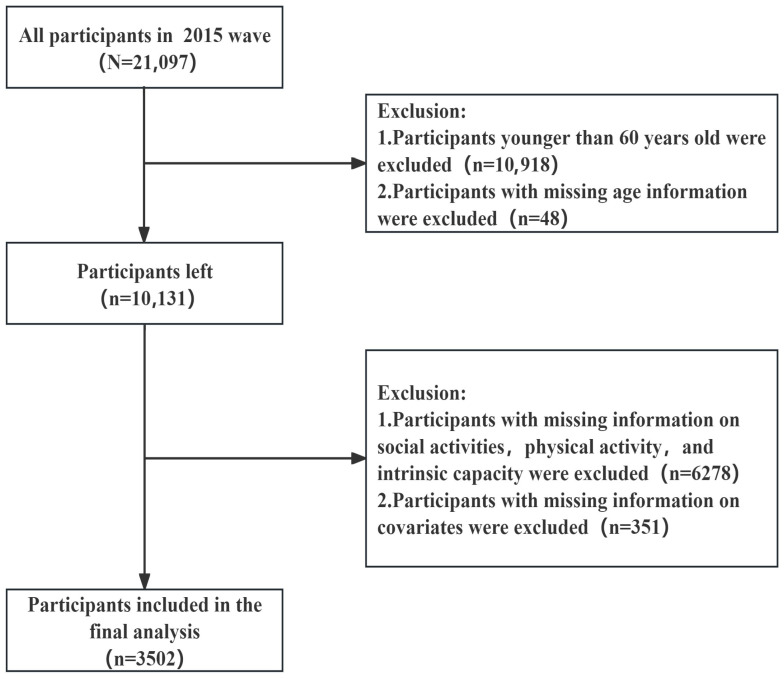
Flowchart of sample selection.

The data from the CHARLS 2015 was utilized for this study. All modules from the 2015 survey that are relevant were merged for analysis. Individuals were excluded if they lacked information on social participation, physical activity, or intrinsic capacity. Furthermore, people under the age of 60 and those lacking information on core covariates were also excluded from the study. The final analytical sample comprised a total of 3502 participants. The detailed flowchart showing participant selection is presented in Figure 1.

### 2.2. Assessment of Social Participation

In the CHARLS questionnaire, social participation (SP) was assessed based on the number and frequency of activities. Participants were asked, “Have you engaged in any of the following social activities in the past month?”, with multiple response options, including: (1) interacting with friends; (2) playing mahjong, cards, or chess, or visiting community activity centers; (3) providing help to family members, friends, or neighbors who do not live together; (4) dancing, fitness, or similar activities; (5) participating in social organizations or clubs; (6) engaging in volunteer or charitable activities; (7) caring for sick or disabled individuals not living in the same household; (8) attending educational or training courses; (9) financial investment activities; (10) internet use; (11) other social activities; and (12) none of the above. One point was awarded for each activity participated in. Option (12) was treated as an independent category; participants selecting this option were assigned a total score of zero. The total activity count ranged from 0 to 11, with higher scores indicating participation in a greater number of activity types.

Participants who selected any of options (1)–(11) were further asked about the frequency of each activity, categorized as (1) almost daily, (2) almost weekly, or (3) not regularly, and assigned values of 3, 2, and 1, respectively. For each activity, the activity score was multiplied by the corresponding frequency score, and all activities were summed to generate a total SP frequency score. The theoretical score range was 0–33, while the observed SP score in this study ranged from 0 to 14. Consistent with previous research [39], SP was categorized into three levels: low (0 points), moderate (1–3 points), and high (>3 points).

### 2.3. Assessment of Physical Activity

To measure participants’ physical activity levels, their level of intensity, days and duration are used. Research places physical activity into three intensity levels with respective metabolic equivalents (MET): vigorous activity (8.0 MET), moderate physical activity (4.0 MET), and light physical activity (3.3 MET). To estimate the daily duration of activity, we used a median value. For instance, the range “≥10 to <30 min” was recorded as 20 min. We took the value for open-ended categories like “≥240 min”. We subsequently computed the weekly PA volume (MET-min/week) as follows: TPA = MET value × daily duration (in minutes) × number of active days per week (in days). Based on the classification criteria established by the International Physical Activity Questionnaire (IPAQ) classification criteria [40], participants were categorized as having a low level (less than 600 MET-min/week), moderate level (600 to 3000 MET-min/week), or high level (>3000 MET-min/week).

### 2.4. Assessment of Intrinsic Capacity

IC is assessed across five dimensions: locomotion, sensory capacity (hearing and vision), vitality, cognition, and psychological capacity. Each dimension received a score of 1 for full functionality and 0 for any impairment. The obtained score can vary from 0 to 5. Higher scores indicated better intrinsic capacity. A score of 4 or lower was defined as IC decline. The criteria for evaluation are shown in Supplementary Table S1.

### 2.5. Covariates

The covariates chosen for this analysis were based on the available literature and the 2015 CHARLS dataset. Factors such as age (60–74/≥75 years), gender (male/female), marital status (partnered/unpartnered), education level (below primary school/primary school/junior middle school/high school and above), residence (urban/rural), currently smoking (yes/no), currently drinking (yes/no), sleep duration (<7 h/≥7 h), number of chronic diseases (0/1/≥2), body mass index (BMI; underweight/normal/overweight/obese), and health insurance (yes/no) were used.

### 2.6. Statistical Analysis

Descriptive statistics were applied to summarize the participants’ traits. Frequencies and percentages served to describe categorical variables. For simple group comparisons, chi-square tests were employed. We utilized logistic regression models to investigate the relationships between the levels of social participation and physical activity (low, moderate, or high) and IC decline. The relationships between the social participation levels and physical activity levels (low, moderate, or high) in relation to IC decline were explored by logistic regression models. Model 1 was unadjusted; Model 2 was adjusted for age, gender, marital status, and education; Model 3 was further adjusted for health behavior-related factors and socioeconomic factors based on the Model 2. The low-level group was set as the reference group. Odds ratios (ORs) with 95% confidence intervals (CIs) and *p*-values were reported for results. Using restricted cubic spline (RCS) models, the potential nonlinear relationships were examined among social participation, physical activity, and IC impairment using nodes at the 5th, 35th, 65th, and 95th percentiles. Several covariates were adjusted in the analyses, including age, sex, marital status, education, residence, currently smoking, currently drinking, sleep duration, number of chronic diseases, BMI, and health insurance. Interaction analysis was conducted to assess the interaction between social participation and physical activity. Stratified analyses also controlled for age, gender, and sleep duration to further assess differences. RCS analysis and subgroup analysis were performed in RStudio 4.4.3, whereas other statistical analyses were performed in STATA 17.0. Two-tailed tests were used for the hypothesis tests and *p*-value < 0.05 was considered statistically significant.

## 3. Results

### 3.1. Baseline Characteristics of the Participants

The fundamental characteristics of the 3502 individuals involved in this research were displayed (Table 1), showing an average age of 67.96 ± 6.47 years. Of the participants, 1758 (50.2%) were male and 1744 (49.8%) were female. Among all participants, 2503 (71.5%) showed an IC decline (average age: 68.45 ± 6.76 years). IC was significantly associated with age, gender, marital status, education, residence, currently smoking, currently drinking, sleep duration, number of chronic diseases, BMI, health insurance, and activity levels (*p* ≤ 0.004). Individuals who were older, female, less educated, unpartnered, living in rural areas, had shorter sleep duration, more chronic diseases, no health insurance, and lower activity levels were more likely to experience IC decline than individuals with normal IC.

To assess potential selection bias, we compared the relevant baseline characteristics between the included group (*n* = 3502) and the excluded group (*n* = 6629); participants who did not meet the study design age criteria were not included in this analysis. As shown in Supplementary Table S2, no statistically significant differences were observed for most covariates (*p* > 0.05).

### 3.2. Association Between Social Participation and Physical Activity Levels and Declines in Intrinsic Capacity

Table 2 indicates that social participation levels are significantly linked to a decline in IC. In the unadjusted Model 1, specifically, mid-to-high-level SP participants had lower IC impairment compared to low SP levels (OR = 0.72, *p* < 0.001; OR = 0.42, *p* < 0.001). These associations continued to be significant following adjustments in Model 3. The mid-to-high-level SP was linked to reduced risks of 20% (OR = 0.80, 95% CI: 0.67–0.95, *p* = 0.012) and 44% (OR = 0.56, 95% CI: 0.45–0.70, *p* < 0.001) as compared to the low-level group. Table 3 indicates that, in Model 3, which was adjusted for all variables, the mid-to-high-level PA compared to the low-level group showed a protective effect on IC, with a reduced risk of IC impairment by 28% (OR = 0.72, 95% CI: 0.55–0.95, *p* = 0.019) and 26% (OR = 0.74, 95% CI: 0.58–0.95, *p* = 0.016), respectively.

We then analyzed the associations between different SP and PA levels and every subdomain of the IC. According to Supplementary Table S3, social participation significantly negatively correlated with locomotion, psychological function, and cognition (*p* < 0.01), and only proved to be statistically significant in the high-level group for sensory function and vitality. We also observed an important connection between physical activity and the locomotion, sensory, and cognitive domains, and moderate links with the vitality domain. Nonetheless, no significant association was observed in the psychological dimension (Supplementary Table S4).

### 3.3. Nonlinear Relationship Between Social Participation and Physical Activity Levels and Intrinsic Capacity Decline

**Figure 2:**
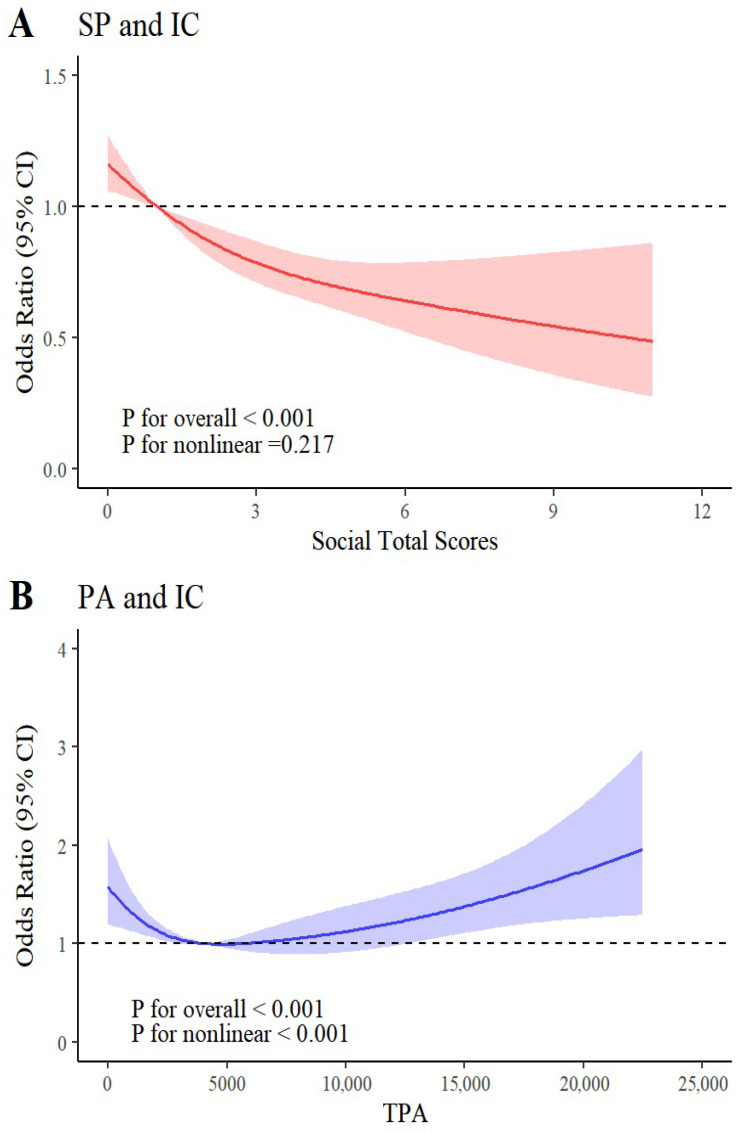
Nonlinear relationship between activity participation levels and risk of intrinsic capacity decline. Note: (**A**) The red solid line represents the estimated odds ratios (ORs) for the risk of IC decline across different levels of social participation, with the red shaded area indicating the corresponding 95% confidence intervals (CIs). (**B**) The blue solid line represents the estimated ORs for the risk of IC decline across different levels of physical activity, with the blue shaded area indicating the corresponding 95% CIs. P for overall and P for nonlinear indicate the significance of the overall association and the nonlinear association, respectively.

To further analyze the relationship between social participation, physical activity, and IC decline, the RCS model was applied to explore potential nonlinear relationships. Figure 2A demonstrates a significant negative dose–response relationship between social participation and the risk of IC impairment. Both the overall trend (*p* < 0.001) and nonlinear values (*p* = 0.217) indicate that the risk of IC decline gradually decreases as the variety and frequency of social activities increase. Figure 2B presents a U-shaped association between physical activity and IC decline risk, with the overall trend and nonlinear values exhibiting statistical significance (all *p* < 0.001). Physical activity levels of approximately 3000–4000 MET-min/week correspond to a comparatively low risk of IC impairment. We assessed the robustness of the results by sequentially including confounding factors. Sensitivity analyses confirmed that the association between social participation, physical activity, and IC decline risk remained robust (Supplementary Figure S1).

Additionally, we further examined the associations of social participation and physical activity with each subdomain of IC. Supplementary Figure S2 reveals significant negative dose–response relationships between social participation and all five dimensions of IC (*p* for nonlinear ≥ 0.496). Meanwhile, Supplementary Figure S3 demonstrates a pronounced nonlinear relationship between physical activity levels and the five IC dimensions (*p* for nonlinear ≤ 0.016).

### 3.4. Interaction Analysis

We further examined the interaction between social participation and physical activity. In the fully adjusted model, the interaction term did not reach statistical significance (*p* for interaction = 0.778), suggesting that there was no significant interaction between social participation and physical activity in their association with intrinsic capacity impairment. Table 4 further presents the predicted risk of intrinsic capacity impairment across different combinations of social participation and physical activity. The results showed that, among all combinations, individuals with high levels of social participation and low levels of physical activity had the lowest predicted risk of intrinsic capacity impairment.

### 3.5. Subgroup Analysis

**Figure 3:**
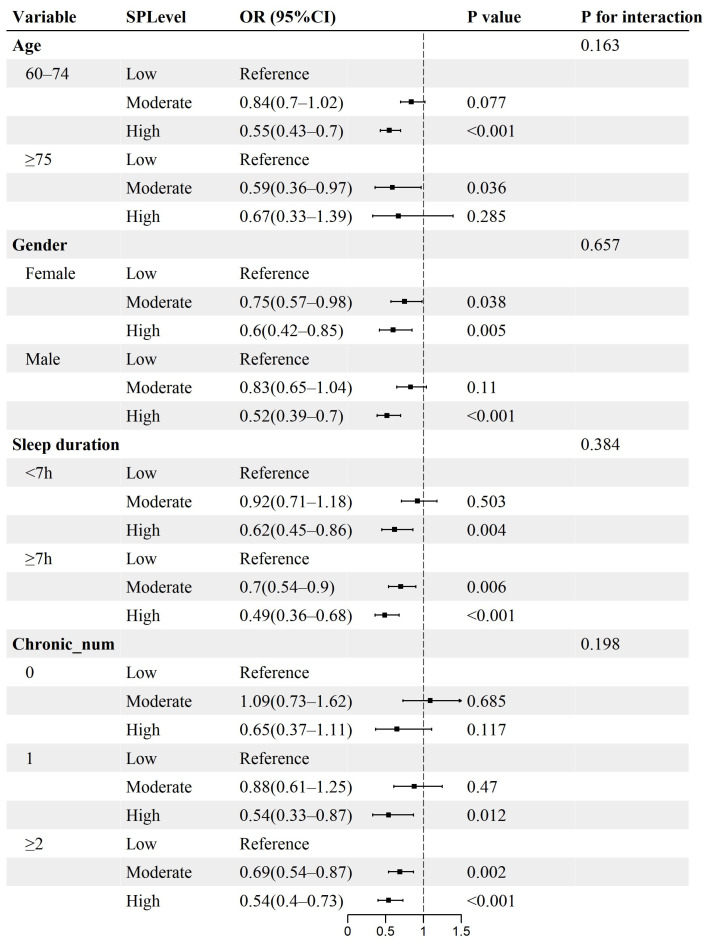
Association between social participation levels and risk of intrinsic capacity decline stratified by different factors. OR, odds ratio; CI, confidence interval. Reference, low-level group.

**Figure 4:**
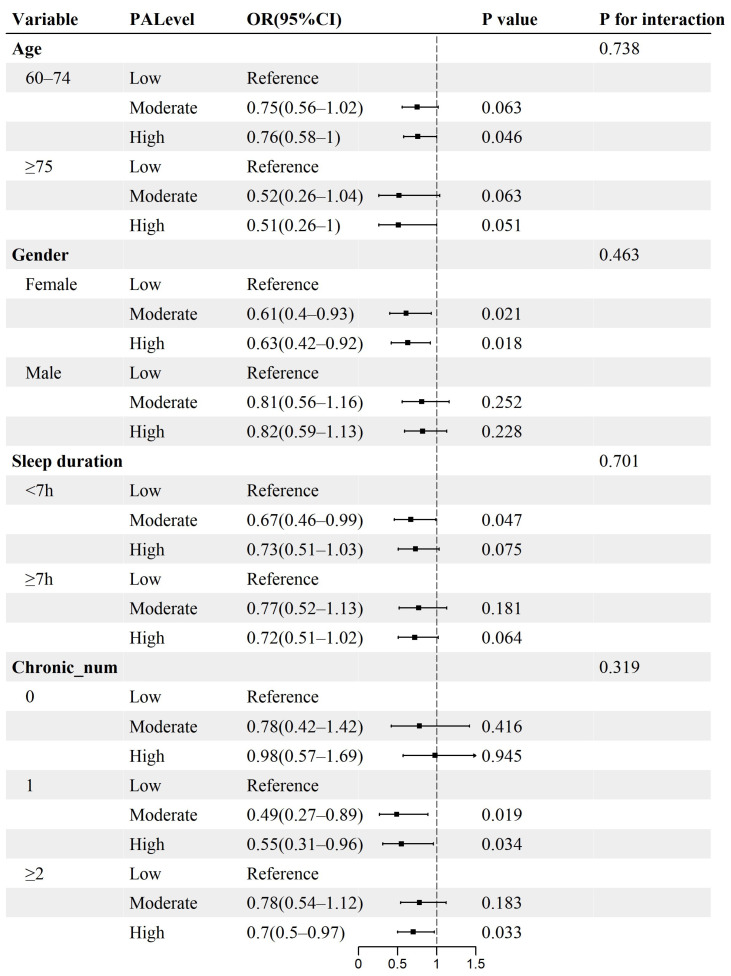
Association between physical activity levels and risk of intrinsic capacity decline stratified by different factors. OR, odds ratio; CI, confidence interval. Reference, low-level group.

Subgroup analyses examined differences in the association between the levels of both SP and PA and IC decline across subgroups. Results showed no significant interactions when stratified by variables such as age, gender, sleep duration, and the number of chronic diseases (Figure 3 and Figure 4). These findings indicate that the association of social participation and physical activity levels with IC decline risk remained consistent across subgroups, with no significant effect modification observed (all *p* values for interaction > 0.05).

## 4. Discussion

This research explored the relationships between both social participation and physical activity and IC decline among older adults. The results suggest that elevated levels of SP and PA are significantly negatively correlated with IC impairment. The RCS model identified a significant negative dose–response relationship between levels of social participation and IC decline risk. Meanwhile, physical activity levels had a U-shaped association with this risk. Interaction analysis revealed no observed interaction between social engagement and physical activity with intrinsic capacity. Analyses of subgroups demonstrated consistent associations across subgroups. These findings underscore that social participation and physical activity may serve as important protective factors for delaying IC decline in later life.

Study results have shown that the incidence of impaired IC in Chinese older adults was relatively high at 71.5%, exceeding the results reported by Zhu et al. (69.6%) [41] and slightly lower than the combined detection rate of IC decline (73.7%) from a recent meta-analysis [42]. Based on the research, it was found that the impairment rates among the elderly in the sensory, psychological, vitality, cognition, and locomotion domains were 41.37%, 35.15%, 23.64%, 19.16%, and 16.13%, respectively. Impairment rates in the sensory, psychological, and vitality domains were higher than those reported by Vinothini et al. [17], while impairment rates in the cognition and locomotion domains were largely consistent. These disparities between the research findings are likely due to differences in study design, sample size, sampling methods, or measurement tools.

Our findings demonstrate a notable negative correlation between SP levels and IC decline risk. Higher SP levels correlate with a reduced risk of IC decline, particularly in the domains of locomotion, psychology, and cognition. This result aligns with earlier studies that have confirmed the positive role of social participation in protecting the functional health of elderly people. Yan et al. found that active involvement in social life can lessen the risk of being limited in actions for people with arthritis [43]. Research by Cheung [44] and Chen et al. [45] showed that participation in social activities not only lowers the risk of dementia and enhances cognitive function but is also associated with a lower risk of depressive symptoms. This connection can be understood from multiple perspectives. Social activities broaden social networks, boost social support, and improve opportunities to access external resources [46]. In the course of interacting with the outside world, the negative psychological impacts resulting from occupational identity transition may be mitigated by enhancing cognitive reserves and achieving self-worth [37] in older adults, such as engaging in community affairs or volunteer activities [47,48,49]. Moreover, involvement in social activities is more likely to sustain independence in ADL of seniors and lower their risk of developing motor–cognitive syndrome [50,51]. At the same time, social interactions usually involve the joint functioning of multiple brain functions, such as communication, thinking, and learning. This cognitive engagement could benefit the preservation of flexibility and reactivity in the brain [52]. Based on earlier studies, if an individual participates in social activities for a long period and is active in them, it may promote a range of healthy behaviors, thereby generating positive health effects across multiple functional domains and reducing the risk of IC decline [53,54]. These findings suggest that strengthening social participation or increasing the accessibility of meaningful activities may help maintain or postpone older people’s IC decline.

This study indicates that physical activity exerts a positive influence on intrinsic capacity, primarily manifested in the domains of exercise, vitality, and cognition. However, unlike the dose–response pattern presented by social participation, PA levels exhibited a U-shaped association with IC decline. This contrasts with earlier findings by Zhou et al. and Luis et al. [34,55,56], who reported a positive relationship between PA and IC—meaning higher levels of PA were linked to better IC. This differs to some extent from previous findings, as our results suggest the greatest benefits for IC are associated with moderate PA, rather than with higher levels, which do not necessarily result in better IC. Aging brings about various physiological changes, including declines in muscle mass, bone density and recovery function [57,58]. Physical exercise is good for postponing loss of muscle strength and bone mineral density. However, individuals vary in how their bodies physiologically adapt to physical activity. When the intensity or duration goes above the physiological tolerance threshold of older adults for exertion, it may cause adverse events such as muscle strains or joint injuries [59], and increase the risk of ADL-limitations. Additionally, some older people objectively lack the physical capacity to engage in high-intensity or prolonged activities [60] due to multiple chronic conditions or other health issues. It may further elevate the probability of exercise-related injuries [61,62,63]. Besides the musculoskeletal system, PA may also affect IC through mechanisms related to the brain health. Inadequate PA may lead to decreased cerebral blood flow and insufficient neurotrophic factor secretion. On the other hand, excessive PA may generate oxidative stress and trigger inflammatory responses within the brain, as reported in other studies [64]. These effects may reduce neural network activity levels [65] and increase IC impairment risk, which aligns with findings from Zhang et al. [66]. Previous studies suggested that moderate PA may enhance balance and cognitive function. In contrast, higher PA may not result in sustained benefits and it could even cause dyspnea and mobility problems [67,68]. To conclude, physical activity contributes immensely to maintaining IC in older adults. One way to promote the health and well-being of elderly people, improve the musculoskeletal system and optimize neuroprotective mechanisms is through moderate exercise [69]. This certainly can provide a basis for public health interventions in an aging society.

The interaction model analysis showed that there was no significant interaction between social participation, physical activity, and IC. However, in the joint distribution of social participation and physical activity, we found that groups characterized by higher social participation generally exhibited a lower overall risk, and the lowest risk of IC decline was observed among individuals with high social participation and low physical activity. This pattern was consistent with the associations suggested by the nonlinear relationship identified in the present study. These findings indicate that social participation may play a more stable and broader role than physical activity in maintaining IC. Intrinsic capacity is not a single indicator of physical function, but rather a multidimensional construct encompassing locomotion, vitality, cognition, psychological well-being, and sensory function. Previous studies have shown that the association between social participation and health in older adults may operate through mechanisms such as social support, social cohesion, and broader community connectedness, and may simultaneously influence both mental and physical health [70,71]. In addition, higher levels of social participation have been associated not only with the maintenance of intact IC, but also with a greater likelihood of intrinsic capacity recovery and a lower risk of decline [14,72]. In contrast, although physical activity is generally beneficially associated with IC, its effects appear to be more domain-specific and context-dependent, with stronger associations observed mainly in specific domains such as locomotion and vitality [34].

The results of the study are robust in subgroup analyses. The study indicated that elevated SP levels were linked to a reduced risk of IC decline. This association was present irrespective of age, gender, sleep duration, and chronic_num subgroups. Nonetheless, elevated levels of PA correlate with a greater risk of IC impairment in the majority of subgroups. Further stratification indicates that there are differences in the association between SP levels and IC decline risk among older adults, according to gender and sleep duration. When SP is at a high level, older men tend to face a lower IC risk than their female counterparts, possibly due to physiological differences between the sexes. Insufficient sleep may also trigger negative activation in regions of the brain that affect cognitive functions related to planning and memory in elderly people [73,74]. Additionally, the connection of PA with the outcome of IC impairment risk appears to be more stable among women and those with a heavier burden of chronic diseases. Women may restrict their physical activity engagement to a greater extent than men due to taking on extra family responsibilities [75]. Moreover, physical pain caused by chronic diseases may limit the daily activity participation of elderly people, which could create an adverse cycle through cumulative tissue damage [76,77]. Thus, appropriately extending sleep duration and strengthening chronic disease management may alleviate the limitations of social and physical activity in older adults, promoting the maintenance of IC and improving quality of life.

As an initial note, this study has certain limitations. The first limitation of the research sample is that it only includes people aged 60 and above in China. Thus, if we summarize the conclusion, the findings may not be generalizable due to demographic characteristics. Moreover, the two main variables, SP and PA levels, were both self-reported through questionnaires. This may suffer from recall bias and social desirability effects. Finally, given that the current study is based on cross-sectional data from CHARLS 2015, its conclusion implies that social participation and physical activity are related to IC decline, and are not necessarily causative. Reverse causation may exist if older adults with poor IC may be less inclined to participate in activities. In addition, the lack of longitudinal data restricts our assessment of dynamic and long-term impacts. Future research should focus on longitudinal studies to further validate the long-term association and potential causal mechanisms between social participation, physical activity, and declines in IC.

## 5. Conclusions

This study examined the relationships between social participation, physical activity, and IC decline. The findings indicated that both social participation and physical activity were independently associated with overall IC and several IC domains in older adults. Older adults with higher levels of social participation had a lower risk of IC decline, while those engaging in moderate physical activity were also less likely to experience IC decline. No significant interaction was observed between social participation and physical activity. However, the joint distribution further suggested that different combinations of social participation and physical activity were associated with differences in IC decline. Compared with physical activity, social participation may play a more stable and broader role in maintaining intrinsic capacity. Therefore, attention to social participation and physical activity intensity in older adults may help delay IC decline and promote healthy aging.

## Figures and Tables

**Table 1:** Participants demographics and baseline characteristics.

Characteristic	Overall *N* = 3502	IC Impairment		χ^2^	*p*-Value
		No *N* = 999	Yes *N* = 2503		
Age, *n* (%)				45.94	<0.001
60–74	2910 (83.1%)	898 (89.9%)	2012 (80.4%)		
≥75	592 (16.9%)	101 (10.1%)	491 (19.6%)		
Gender, *n* (%)				89.65	<0.001
Female	1744 (49.8%)	371 (37.1%)	1373 (54.9%)		
Male	1758 (50.2%)	628 (62.9%)	1130 (45.1%)		
Marital status, *n* (%)				34.69	<0.001
Partnered	2861 (81.7%)	877 (87.8%)	1984 (79.3%)		
Unpartnered	641 (18.3%)	122 (12.2%)	519 (20.7%)		
Education, *n* (%)				219.09	<0.001
Below primary school	2029 (57.9%)	391 (39.1%)	1638 (65.4%)		
Primary school	806 (23.1%)	300 (30.0%)	506 (20.2%)		
Middle school	450 (12.8%)	200 (20.0%)	250 (10.0%)		
High school and above	217 (6.2%)	108 (10.8%)	109 (4.4%)		
Residence, *n* (%)				72.53	<0.001
Rural	2290 (65.4%)	545 (54.6%)	1745 (69.7%)		
Urban	1212 (34.6%)	454 (45.4%)	758 (30.3%)		
Currently smoking, *n* (%)				8.40	0.004
No	2513 (71.8%)	682 (68.3%)	1831 (73.2%)		
Yes	989 (28.2%)	317 (31.7%)	672 (26.8%)		
Currently drinking, *n* (%)				40.44	<0.001
No	2368 (67.6%)	596 (59.7%)	1772 (70.8%)		
Yes	1134 (32.4%)	403 (40.3%)	731 (29.2%)		
Sleep duration, *n* (%)				26.92	<0.001
˂7 h	1921 (54.9%)	479 (47.9%)	1442 (57.6%)		
≥7 h	1581 (45.1%)	520 (52.1%)	1061 (42.4%)		
Chronic_num, *n* (%)				69.15	<0.001
0	554 (15.8%)	231 (23.1%)	323 (12.9%)		
1	811 (23.2%)	251 (25.1%)	560 (22.4%)		
≥2	2137 (61.0%)	517 (51.8%)	1620 (64.7%)		
BMI, *n* (%)				38.21	<0.001
Normal weight	1780 (50.8%)	496 (49.6%)	1284 (51.3%)		
Underweight	268 (7.7%)	41 (4.1%)	227 (9.1%)		
Overweight	1065 (30.4%)	359 (35.9%)	706 (28.2%)		
Obese	389 (11.1%)	103 (10.3%)	286 (11.4%)		
Health insurance, *n* (%)				12.67	<0.001
No	292 (8.3%)	57 (5.7%)	235 (9.4%)		
Yes	3210 (91.7%)	942 (94.3%)	2268 (90.6%)		
SP level, *n* (%)				71.13	<0.001
Low level	1730 (49.4%)	406 (40.6%)	1324 (52.9%)		
Moderate level	1252 (35.8%)	373 (37.3%)	879 (35.1%)		
High level	520 (14.8%)	220 (22.0%)	300 (12.0%)		
PA level, *n* (%)				22.23	<0.001
Low level	539 (15.4%)	109 (10.9%)	430 (17.2%)		
Moderate level	911 (26.0%)	283 (28.3%)	628 (25.1%)		
High level	2052 (58.6%)	607 (60.8%)	1445 (57.7%)		

**Table 2:** Association between social participation levels and intrinsic capacity decline.

Variables	Social Participation	Model 1 OR (95%CI)	*p*-Value	Model 2 OR (95%CI)	*p*-Value	Model 3 OR (95%CI)	*p*-Value
Social participation	Low level	REF		REF		REF	
	Moderate level	0.72 (0.61, 0.85)	<0.001	0.78 (0.66, 0.93)	0.005	0.80 (0.67, 0.95)	0.012
	High level	0.42 (0.34, 0.51)	<0.001	0.53 (0.43, 0.66)	<0.001	0.56 (0.45, 0.70)	<0.001

Note: Model 1 was a crude model. Model 2 is adjusted for age, gender, marital status and education. Model 3 further adjusted for currently smoking, currently drinking, sleep duration, chronic_num, BMI, residence and health insurance based on Model 2.

**Table 3:** Association between physical activity levels and intrinsic capacity decline.

Variables	Physical Activity	Model 1 OR (95%CI)	*p*-Value	Model 2 OR (95%CI)	*p*-Value	Model 3 OR (95%CI)	*p*-Value
Physical activity	Low level	REF		REF		REF	
	Moderate level	0.56 (0.44, 0.72)	<0.001	0.65 (0.50, 0.85)	0.001	0.72 (0.55, 0.95)	0.019
	High level	0.60 (0.48, 0.76)	<0.001	0.72 (0.56, 0.91)	0.007	0.74 (0.58, 0.95)	0.016

Note: Model 1 was a crude model. Model 2 is adjusted for age, gender, marital status and education. Model 3 further adjusted for currently smoking, currently drinking, sleep duration, Chronic_num, BMI, residence and health insurance based on Model 2.

**Table 4:** Predictive margins of intrinsic capacity impairment across combined categories of social participation and physical activity.

Groups	Delta-Method					
	Margin	Std. Err.	z	*p* > |z|	[95% Conf. Interval]	
SP level * PA level						
Low level * Low level	0.7779	0.0184	42.39	0.000	0.7420	0.8139
Low level * Moderate level	0.7435	0.0206	36.01	0.000	0.7030	0.7840
Low level * High level	0.7338	0.0135	54.51	0.000	0.7074	0.7601
Moderate level * Low level	0.7397	0.0311	23.78	0.000	0.6788	0.8007
Moderate level * Moderate level	0.6859	0.0237	28.97	0.000	0.6395	0.7323
Moderate level * High level	0.7079	0.0161	43.89	0.000	0.6763	0.7395
High level * Low level	0.5591	0.0908	6.16	0.000	0.3812	0.7370
High level * Moderate level	0.6213	0.0429	14.50	0.000	0.5373	0.7053
High level * High level	0.6312	0.0268	23.62	0.000	0.5789	0.6836

Note: Confounders included age, gender, marital status, education, residence, currently smoking, currently drinking, sleep duration, Chronic_num, BMI and health insurance.

## Data Availability

This research relies on datasets that are accessible to the public. The data utilized and examined in this research originates from CHARLS (http://charls.pku.edu.cn (accessed on 15 September 2025)), which is a longitudinal survey that represents the nation and is carried out by the Institute of Social Science Surveys at Peking University.

## Supplementary Materials

The following supporting information can be downloaded at https://www.mdpi.com/article/10.3390/healthcare14070936/s1: Table S1: Intrinsic capacity evaluation standard; Table S2: Baseline characteristics of participants excluded and retained in the study; Table S3: Associations between social participation and five subdomains of intrinsic capacity; Table S4: Associations between physical activity and five subdomains of intrinsic capacity; Figure S1: Nonlinear relationships between social participation and physical activity levels and intrinsic capacity decline; Figure S2: Nonlinear relationships between social participation levels and five subdomains of intrinsic capacity; Figure S3: Nonlinear relationships between physical activity levels and five subdomains of intrinsic capacity.
