# Supplementary material for: Association Between Social Participation, Physical Activity, and Intrinsic Capacity Decline: Empirical Evidence from the CHARLS
Source: Healthcare (Basel). 2026 Apr 3;14(7):936. doi: 10.3390/healthcare14070936 (PMC13073356; doi:10.3390/healthcare14070936)
Supplement: Supplementary file 1 [file healthcare-14-00936-s001.zip › Revised Supplementary Documents.pdf]

Supplementary Material

Association between Social Participation, Physical Activity, and Intrinsic Capacity Declines: Empirical Evidence from the CHARLS

Supplementary Table S1.Intrinsic capacity evaluation standard. 2
Supplementary Table S2. Baseline characteristics of participants excluded and retained in the study 3
Supplementary Table S3. Associations between social participation and five subdomains of intrinsic capacity. 5
Supplementary Table S4. Associations between physical activity and five subdomains of intrinsic capacity. 6
Supplementary Figure S1. Nonlinear relationships between social participation and physical activity levels and intrinsic capacity decline. 7
Supplementary Figure S2. Nonlinear relationships between social participation levels and five subdomains of intrinsic capacity. 8
Supplementary Figure S3. Nonlinear relationships between physical activity levels and five subdomains of intrinsic capacity. 9

Supplementary Table S1.Intrinsic capacity evaluation standard.

Dimension	Evaluation Standards
Locomotion	Locomotion was assessed using the Short Physical Performance Battery (SPPB), which includes a 4 m walking speed test, standing balance tests at three positions, and a five-time chair stand test. The total SPPB score ranges from 0 to 12, with a score <9 indicating impaired locomotion.
Sensory	Sensory capacity was determined based on self-reported hearing and vision status. Participants reporting "poor" hearing or vision were considered to have sensory impairment.
Vitality	Vitality was assessed using handgrip strength. Grip strength <28 kg for men and <18 kg for women was defined as impaired vitality.
Cognitive	Cognitive capacity was evaluated using the modified Chinese version of the Mini-Mental State Examination (MMSE), covering four domains: orientation (0–5 points), memory (average score of immediate and delayed recall of 10 Chinese words; 0–10 points), calculation (serial subtraction of 7 from 100; 0–5 points), and visuospatial ability (accurate figure drawing; 0–1 point). The total cognitive score ranged from 0 to 21, with lower scores indicating poorer cognitive function. Cognitive impairment was defined as a score more than one standard deviation below the age-specific mean (grouped in 5-year intervals).
Psychological	Psychological capacity was assessed using the 10-item Center for Epidemiologic Studies Depression Scale (CES-D-10). Scores range from 0 to 30, with higher scores indicating more severe depressive symptoms. A CES-D-10 score ≥10 was defined as psychological impairment.

Supplementary Table S2. Baseline characteristics of participants excluded and retained in the study

Variable	retained participants	excluded participants	χ^2	*P* value
Number	3502	6629		
Age, *n* (%)			3.236	0.072
60-74	2,910 (83.1%)	5413 (81.7%)		
≥75	592 (16.9%)	1216(28.3%)		
Gender, *n* (%)			1.756	0.185
Male	1,758 (50.2%)	3,236 (48.8%)		
Female	1,744 (49.8%)	3,393 (51.2%)		
Marital status, *n* (%)			3.522	0.061
With spouse	2,861 (81.7%)	5,305 (80.0%)		
Without spouse	641 (18.3%)	1,314 (20.0%)		
Education, *n* (%)			17.817	<0.001
Below Primary School	2,029 (57.9%)	3,703 (55.9%)		
Primary School	806 (23.1%)	1,487 (22.4%)		
Middle School	450 (12.8%)	875 (13.2%)		
High School and above	217 (6.2%)	562 (8.5%)		
Residence, *n* (%)			62.881	<0.001
Rural	2,290 (65.4%)	3,797 (57.3%)		
Urban	1,212 (34.6%)	2,832 (42.7%)		
Currently smoking, *n* (%)			1.998	0.158
No	2,513 (71.8%)	4,789 (73.1%)		
Yes	989 (28.2%)	1,767 (26.9%)		
Currently drinking, *n* (%)			0.660	0.417
No	2,368 (67.6%)	4,485 (68.4%)		
Yes	1,134 (32.4%)	2,071 (31.6%)		
Sleep duration, *n* (%)			0.826	0.363
<7h	1,921 (54.9%)	3,061 (53.9%)		
≥7h	1,581 (45.1%)	2,620 (46.1%)		

Chronic_num, *n* (%)			12.923	0.002
0	554 (15.8%)	900 (13.6%)		
1	811 (23.2%)	1,468 (22.1%)		
≥2	2,137 (61.0%)	4,261 (64.3%)		
BMI, *n* (%)			6.736	0.081
Normal weight	1,780 (50.8%)	2,344 (51.8%)		
Underweight	268 (7.7%)	403 (8.9%)		
Overweight	1,065 (30.4%)	1,287 (28.4%)		
Obese	389 (11.1%)	495 (10.9%)		
Health insurance, *n*（%）			1.182	0.277
No	292 (8.3%)	533 (9.0%)		
Yes	3,210 (91.7%)	5,394 (91.0%)		

Supplementary Table S3. Associations between social participation and five subdomains of intrinsic capacity.

Variables	Locomotion OR (95%CI)	Sensory OR (95%CI)	Vitality OR (95%CI)	Cognition OR (95%CI)	Psychological OR (95%CI)
Low level	REF	REF	REF	REF	REF
Medium level	0.73** (0.59,0.90)	0.89 (0.76,1.03)	0.86 (0.72,1.02)	0.70** (0.57,0.86)	0.81* (0.69,0.95)
High level	0.55*** (0.40,0.76)	0.70** (0.56,0.86)	0.62** (0.47,0.81)	0.46*** (0.32,0.65)	0.60*** (0.48,0.76)

Note: * p<0.05, ** p<0.01,*** p<0.001. Confounders included age, gender, marital status, education, residence,currently smoking, currently drinking, sleep duration, Chronic_num, BMI and health insurance.

Supplementary Table S4. Associations between physical activity and five subdomains of intrinsic capacity.

Variables	Locomotion OR (95%CI)	Sensory OR (95%CI)	Vitality OR (95%CI)	Cognition OR (95%CI)	Psychological OR (95%CI)
Low level	REF	REF	REF	REF	REF
Medium level	0.75* (0.58,0.98)	0.77* (0.61,0.96)	0.86 (0.61,1.10)	0.64** (0.48,0.86)	0.81 (0.64,1.03)
High level	0.44*** (0.34,0.56)	0.75** (0.61,0.91)	0.59*** (0.47,0.74)	0.71** (0.56,0.92)	0.92 (0.74,1.13)

Note: * p<0.05, ** p<0.01,*** p<0.001. Confounders included age, gender, marital status, education, residence,currently smoking, currently drinking, sleep duration, Chronic_num, BMI and health insurance.

Supplementary Figure S1. Nonlinear relationships between social participation and physical activity levels and intrinsic capacity decline.

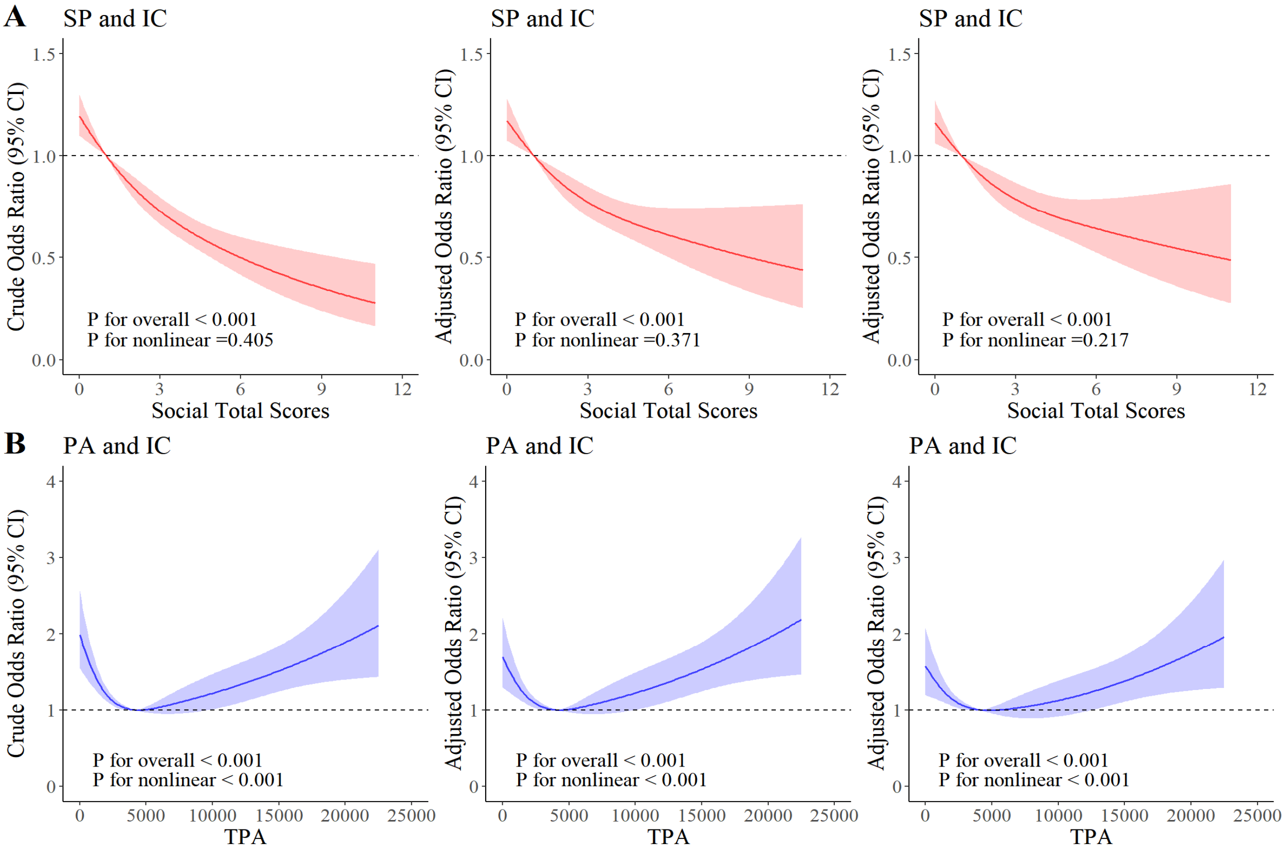

Note: Model 1 was a crude model. Model 2 is adjusted for age, gender, marital status and education.Model 3 further adjusted for currently smoking, currently drinking, sleep duration, chronic_num, BMI, residence and health insurance based on Model 2.

Supplementary Figure S2. Nonlinear relationships between social participation levels and five subdomains of intrinsic capacity.

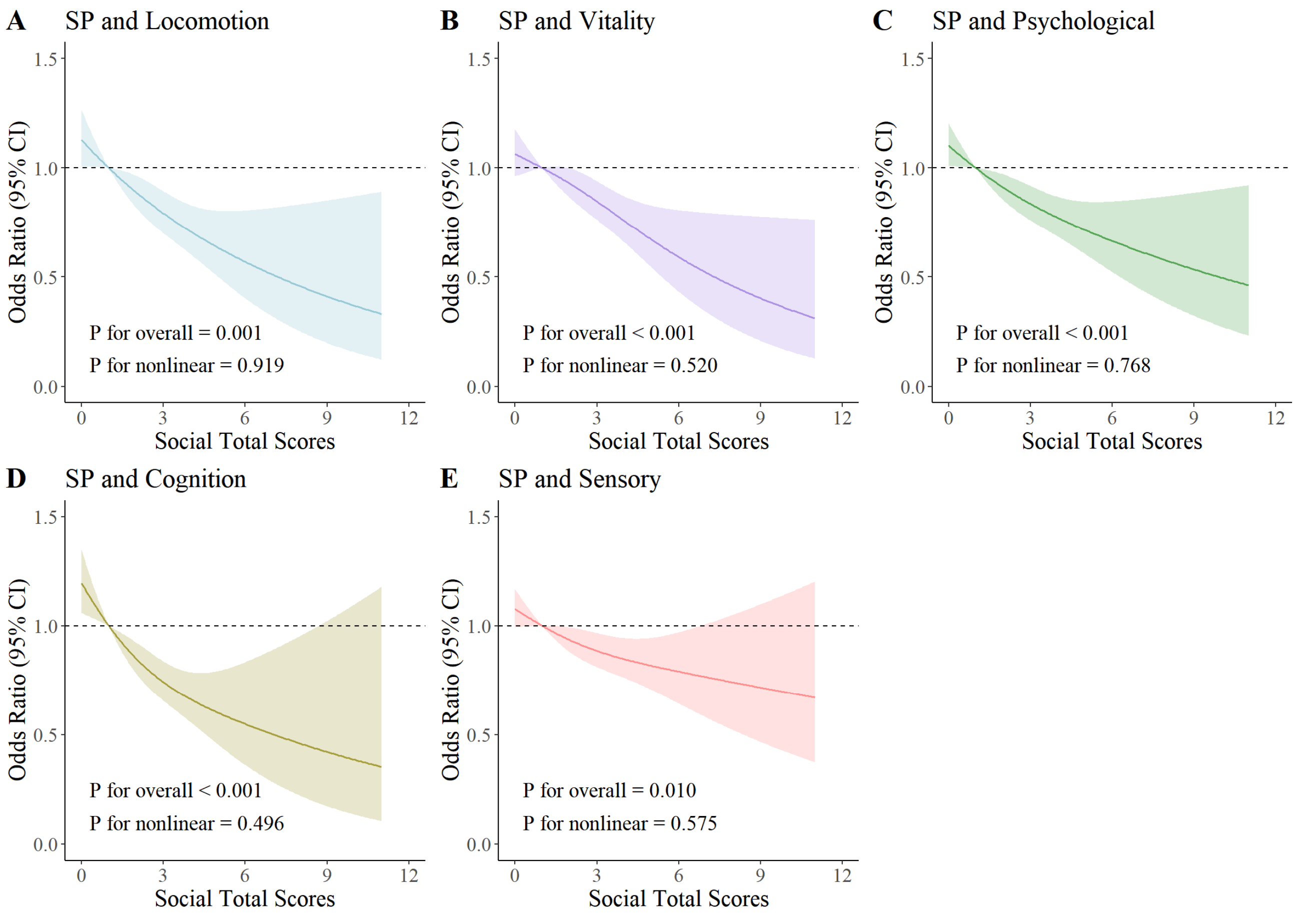

Note:Confounders included age, gender, marital status, education, residence,currently smoking, currently drinking, sleep duration, chronic_num, BMI and health insurance.

Supplementary Figure S3. Nonlinear relationships between physical activity levels and five subdomains of intrinsic capacity.

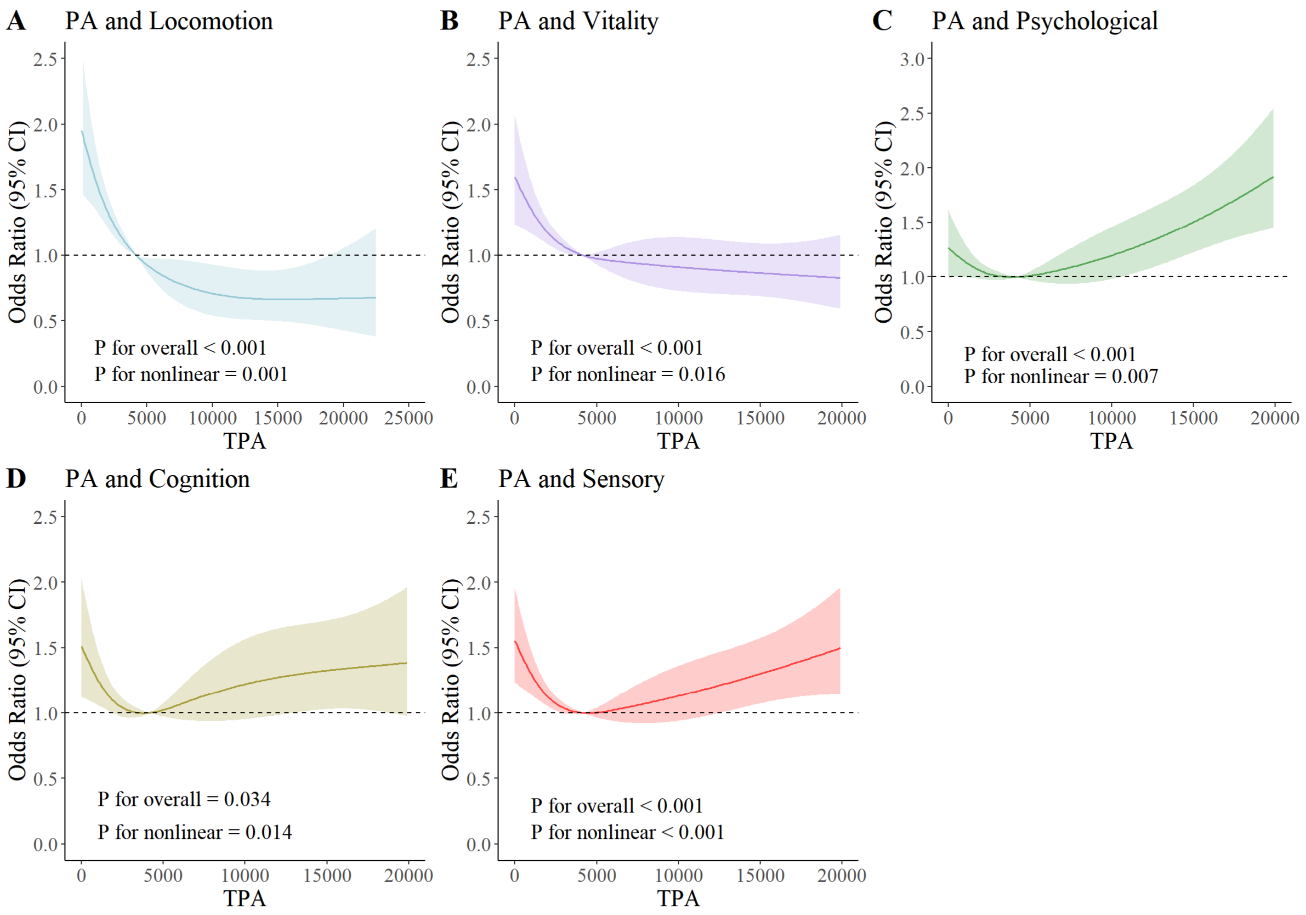

Note: Confounders included age, gender, marital status, education, residence,currently smoking, currently drinking, sleep duration, chronic_num, BMI and health insurance.
